# Assessing post-cold challenge recovery of thermography as a potential outcome measure in trials of SSc-related Raynaud’s phenomenon

**DOI:** 10.1038/s41598-026-50510-5

**Published:** 2026-05-14

**Authors:** Abigail Aika Ndosi, Graham Dinsdale, Joanne Manning, Melissa Mandzuk, Sarah Wilkinson, Ariane L. Herrick, Andrea K. Murray

**Affiliations:** 1 Scleroderma and Raynaud’s Research Group, Centre for Musculoskeletal Research, Faculty of Biology Medicine and Health, The University of Manchester, Manchester, UK; 2 Department of Rheumatology, Salford Care Organisation, Northern Care Alliance NHS Foundation Trust, Salford, UK; 3 NIHR Manchester Biomedical Research Centre, Manchester Academic Health Science Centre, Manchester University NHS Foundation Trust, Manchester, UK

**Keywords:** Thermal imaging, Scleroderma, Raynaud’s phenomenon, Clinical trials outcome measures, Feasibility, Immunology, Physiology, Biomarkers, Diseases, Medical research, Rheumatology

## Abstract

Improved new medications for systemic sclerosis-related Raynaud’s phenomenon (SSc-RP) are badly needed and better objective outcomes are required to enable this. Thermography imaging of hand cold-challenge offers potential as an outcome measure. To ensure standardisation between pre- and post-dose cold challenge assessments, it is important to understand the length of time required for patients to recover back to baseline finger temperatures after initial cold challenge. The aim of this study was to determine whether patients with SSc-RP would recover baseline finger temperatures within a 2-h period. Patients with SSc-RP underwent cold-challenge alongside thermography-imaged baseline and post-cold challenge recovery for 2 h. Twenty patients were recruited, eight (40%) had all eight fingers recover to baseline within 2 h, 13 (65%) had more than one finger recover. The median (IQR) time to maximum temperature was 48.43 (33.58–69.38) minutes. This study indicates that most patients will have one or more fingers recover within an hour. Even in those with established SSc it is reasonable to repeat cold challenge with a minimum one-hour interval in clinical trials, enabling pre- and post-dose cold challenges on the same day.

**Supplementary Information:**

The online version contains supplementary material available at 10.1038/s41598-026-50510-5.

## Introduction

Systemic sclerosis (SSc) is a connective tissue disease associated with vasculopathy, over 95% of patients experience Raynaud’s phenomenon (RP)^1^, which is often the presenting feature and can be very severe. However, RP affects approximately 5% of the otherwise healthy population^2^, termed ‘primary’ (idiopathic) RP. Thermography-monitored cold challenge can be used as part of the diagnostic process to differentiate primary from SSc-related RP (SSc-RP), and it is also beginning to show utility as an outcome measure for clinical trials of vasodilators^3–9^.

Clinical trials of SSc-RP have often failed to meet their primary outcome with a suggested explanation being the use of subjective patient-reported outcome measures, which may not be sensitive to change. Better outcome measures would potentially facilitate clinical trials with lower numbers of participants (higher power)^10^.

We have previously shown that response to a cold challenge, as assessed by either thermography or by laser speckle contrast imaging, holds promise as a reliable outcome measure^11^, but sensitivity to change has yet to be established. To determine sensitivity to drug-induced change, for example in the context of an early phase proof-of-concept study, cold challenges are required at least pre and post dosing. The minimum time interval between these cold challenges (to ensure that the participant’s hands have returned to baseline following the first cold challenge) has not been investigated. Determining how long patients require to recover (rewarm) to baseline temperature would inform the practical number of cold challenges possible within one day as part of a trial protocol.

The aim of this study was to better understand how patients recover beyond a standard (clinical diagnostic) 15-min rewarming. Specific objectives were to test the hypotheses that 1) the majority of patients with SSc-related RP recover within a 2-h period, and 2) that initial 15-min recovery measurements are related/predictive of 2-h recovery. Further objectives were to understand more about the relationships between the temperature of the hands before and immediately after cold challenge in those that did and did not recover and whether baseline finger/hand temperatures could predict time to recovery.

## Material and methods

Adult patients (over 18 years of age) with a confirmed diagnosis of SSc (according to the ACR 2013 criteria^12^) or fulfilling the criteria of Very Early Diagnosis Of SSc (VEDOSS)^13^, with stable medication for at least 1 month prior to their visit (including no prostacyclin analogue treatment within the last month) were recruited and attended for a single visit. Inclusion criteria stated an ability to provide signed informed consent and a history of RP defined as digital cold sensitivity associated with colour changes (cyanosis and pallor). Exclusion criteria included lack of capacity to consent, active ulcers or finger contractures (which would preclude cold challenge and subsequent imaging), recent treatment with prostacyclin as well as diabetes or other circulatory conditions. Patients were asked to avoid caffeine and nicotine for 4 h prior to their visit. Imaging was carried out in a temperature-controlled room (23 ± 2 °C). Images were taken with a FLIR T540 thermal camera. All patients gave written informed consent. The study was approved by West London & GTAC Research Ethics Committee. All methods were performed in accordance with the relevant guidelines and regulations.

**Fig. 1:**
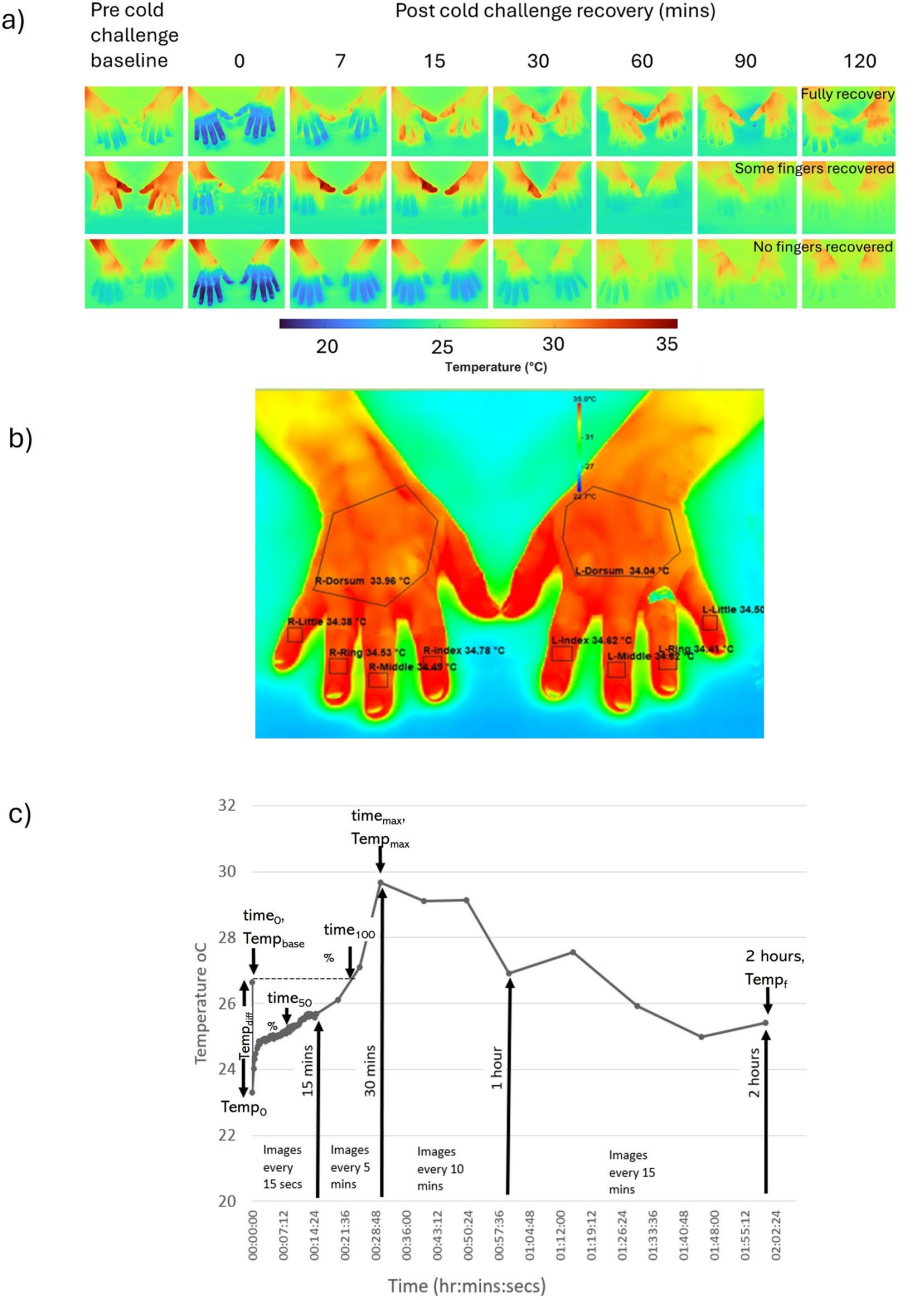
(**a**) Series of frames showing the thermal image of hand at baseline, immediately post cold challenge (0 min) and then at times labelled above the frames to 2 h (120 mins). All images are on the same colour scale with the scale bar below the images The top line is an example of a participant who fully recovered, the middle line shows a participant in whom some fingers recovered, and the bottom line shows images from a participant who had no fingers recover. (**b**) A baseline image showing the regions of interest chosen, at the distal fingers and dorsum of the hand, to calculate the distal dorsal difference (DDD) and the finger temperature over time, (**c**) An example of one participant’s recovery curve averaged for all 8 fingers, annotated to show times and temperature points of interest. Annotations show: the temperature at baseline Temp_base_, at time 0, time_0_ (before cold challenge); Temp_0_, Initial post-cooling temperature; Temp_diff_, difference between baseline and post cooling temperature; Temp_max_, maximum temperature; time_max_, at time to maximum temperature; Temp_f_, final temperature, at 2 h; time_50%_, time to 50% recovery; time_100%_, time to 100% recovery.

Patients were initially seated and acclimatised to the room conditions for 20 min. Both hands were then imaged to provide a baseline image of hand temperature. A cold challenge was then performed, both hands (nitrile-gloved) were submerged in 15°C water to the metacarpophalangeal joints for 1 min. The hands were removed from water, gloves discarded, and post-cold challenge imaging commenced immediately. Thermal images of both hands together were taken every 15 s (to 15 min), then every 5 min (to 30 min), then every 10 min (to 60 min), and finally every 15 min (to 120 min). For the first 15 min patients were asked to remain seated and still. Patients were allowed to move for comfort between images after 15 min. Imaging was stopped at 120 min even if patients had not fully recovered (Fig. 1a).

Images were analysed to extract skin temperature in relevant regions of interest (8 distal fingers and the dorsa of the hands) using bespoke software (ThermographySRFT version 2.1.0). From baseline images, for each finger, baseline temperature (Temp_base_) was extracted and distal dorsal difference (DDD: temperature difference between the dorsum of the hand and the distal finger) was calculated (Fig. 1b).

Figure 1c shows an example of a recovery curve, annotated to show outcome measures. Maximum temperature achieved (Temp_max_) was compared to baseline (Temp_base_)_,_ and time to maximum temperature (time_max_) was calculated. Finger temperature difference was calculated between the temperature immediately post cold challenge (Temp_0_), baseline pre-cold challenge, (Temp_base_-Temp_0_ = Temp_diff_). Area under the recovery curve (AUC), and final temperature (Temp_f_) were extracted. Time to 25% (time_25%_), 50% (time_50%_), 100% (time_100%_) recovery of baseline pre-cold challenge were measured. For those that did not recover, time_100%_ was set to 2 h.

All data was averaged over 8 fingers and analysed in MATLAB (V2019a). Cox regression assessed the relationship between T_base_, DDD and time to recovery (time_100%_) for the whole group. For the tables and figures, data was split into three groups: those that had all 8 fingers recover, those who had between 1 and 7 fingers recover, and those that did not have any fingers recover. Due to the small numbers in these groups only descriptive data were generated.

## Results

**Fig. 2:**
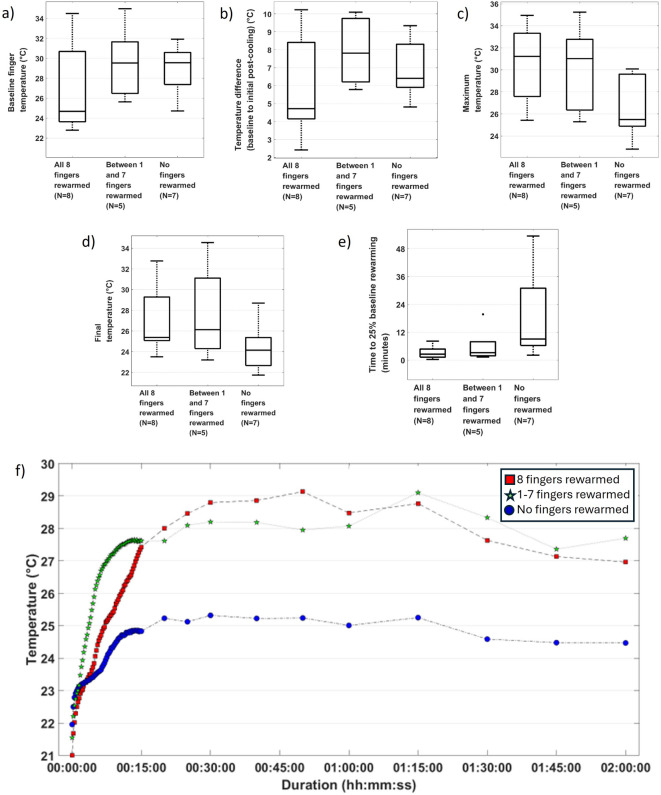
Box Plots of finger temperature (**a**) Temp_baseline_, (**b**) the temperature difference between baseline and initial post-cooling temperature (Temp_diff_), (**c**)Temp_max_ and (**d**) Temp_f_ (^o^C) and (**e**) time to 25% (time_25%_) of baseline recovery for each group (all fingers recovered, more than one 1-7 fingers recovered and no fingers recovered), (**f**) Recovery curves (mean of all 8 fingers per person, mean per group) for those in whom all 8 fingers recovered (N = 8, red squares), those who had between 1 and 7 fingers recover (N = 5, green stars) and those who had no fingers recover (N = 7, blue circles).

Twenty participants with SSc were recruited; demographics and clinical characteristics are shown in supplementary Table 1. Temperature and time data are shown in supplementary Table 2 and Fig. 2.

### Hypothesis 1: that most patients with SSc-related RP recover within a 2-h period

Eight patients (40%) had all eight fingers recover to baseline within 2 h; 13 (65%) had more than one finger recover (eight had eight fingers, one had one, one had four, one had six and two had seven fingers recover). The median (interquartile range [IQR]) time to maximum temperature was 48.43 (33.58–69.38) minutes, after which finger temperature decreased. The longest time to recover for any finger within the 2-h period was 110 min (for a patient that only had one finger recover).

### Hypothesis 2: that initial 15-min recovery measurements are predictive of 2-h recovery profile

Times to percentage of baseline recovery, time_25%,_ and time_50%_, were shorter in the fully recovered group than the non-recovered group. In the eight-finger recovered cohort, all eight patients (100%) achieved time_25%_ within the first 15 min and seven (88%) achieved time_50%_ within 15 min. In those that had at least one finger recover in 2 h, 12 out of 13 (92%) achieved time_25%_ and ten (77%) achieved time_50%_ within 15 min. For the non-recovered group, 3 out of 7 (43%) achieved time_25%_ and 2 (29%) achieved time_50%_ within 15 min. The difference in the recovery trends between groups can clearly be seen in Fig. 2f.

### Relationships between the temperature of the hands before and immediately after cold challenge in those that did and did not recover

In the cohort of participants that had eight fingers recover, pre-cooling, Temp_base_ was lower than those who did not recover (although the spread was large, Fig. 2a); colder fingers led to a larger difference between fingers and the dorsum (and thus a larger DDD). Post cooling, Temp_0_ was lower in those that recovered than those who had no fingers recover and Temp_diff_ (the initial drop in temperature post cold challenge) was smaller, (i.e. in those that fully recovered, finger temperature decreased less than for those who did not recover, Fig. 2b). Temp_max_, and Temp_f_ were higher in the group who had eight fingers recover than in the group with no fingers recovered (supplementary Table 2, Fig. 2c and d). Figure 2f shows the mean recovery profiles for patients, with distinct recovery curve patterns. For those that recover (one or more fingers) there is a steep gradient within the first 10–15 min. For those that do not recover the gradient decreases after approximately 1 min. By 20 min the gradient of recovery has slowed for all three groups but for the non-recovered group the temperature reached is 2–3 °C less than for those that recovered.

Cox regression showed a weakly negative relationship between Temp_base_ and time_100%_ (β = −1.07, Standard error (SE) = 0.60, *p* = 0.07). DDD showed no relationship to time_100%_ (β = −0.61, Standard error (SE) = 0.88, *p* = 0.49)_._ Thus, finger temperature before cold challenge is unlikely predict time to recovery.

## Discussion

### Hypothesis 1: that most patients with SSc-related RP recover within a 2-h period

The majority of patients had one or more fingers recover to baseline temperatures in the 2-h period, indicating the possibility of measuring improvement with a new treatment. There were three distinct groups; those that fully recovered (N = eight patients, 40%), those that had some fingers recover (N = 13, 65%) and those that had no fingers recover (N = seven, 35%). The median (IQR) time to maximum temperature was 48.43 (33.58–69.38) minutes. In those who had fully recovered, cooling occurred towards the end of the two-hour period; sitting for long periods prohibits further recovery and may be detrimental to recovery to baseline in some patients.

### Hypothesis 2: that initial 15-min recovery measurements are predictive of 2-h recovery profile

Data indicates that early initiation of recovery leads to full rewarming. If a patient reached time_50%_ within 15 min they were highly likely to recover fully over 2 h. The likelihood of longer-term recovery could be predicted by the initial 15-min recovery; thus screening may identify patients who will fully recover. This is also observed in Fig. 2f. It is possible that those in whom no fingers recovered, there was less ability to vasodilate, perhaps due to more severe underlying structural disease.

### Relationships between the temperature of the hands before and after cold challenge in those that did and did not recover

In the group that fully recovered the baseline temperature (Temp_base_) was lower (and DDD larger), requiring less recovery. Neither Temp_base_ nor DDD predicted time to recovery (time_100%_). The three groups had discreet recovery trajectories, with a lower gradient of recovery in those who did not recover (Fig. 2f).

Thermography has been used as an indirect outcome measure of finger perfusion in many observational studies and more recently in clinical trials^3–9^. One difficulty in comparing studies has been different cooling protocols and thermographic parameters/outcomes. Standardising protocols, including of measured parameters, would enable better comparisons between trials. The SSc community continue to make progress in this area^11,14–17^. Establishing whether multiple cold challenges can be given on the same day (pre and post treatment) has the potential of allowing more precise monitoring of drug pharmacokinetics and response to cold challenges.

Of the cohort that fully recovered, all achieved this within the first hour, potentially meaning that the window between cold challenges could be less than two hours. This study indicates that monitoring recovery post-cold challenge for 15-min will aid screening for those patients, who will respond to cold challenge within shorter time frames (< 1 h) and offers a route to pre-select patients for studies to enable two cold challenges in a day and make Phase 2 study design more streamlined.

When designing a trial protocol, a tolerance on the temperature recovered by patients (e.g. to within 90% of baseline may be acceptable) and the number of fingers recovered needs to be considered in terms of how to categorise a patient as ‘recovered/rewarmed’.

The main limitation of the study is the small sample size, particularly when in subsets; however, even in this study it is worth noting within this cohort of patients, the group that fully recovered had higher (more severe) patient reported outcome measures (RCS^18^) than those who did not recover. This is indicative that those patients who fully recovered had lower baseline temperatures, but this also indicates a divergence between subjective patient reported outcome measures and thermography^17^.

In multicentre studies, particularly international trials, or those run over extended periods of time, it may be necessary to consider the effects of seasonal variations and local environmental conditions. Whilst some work has been done in this area, a greater understanding is still required^18–21^.

This study provides insight into the longer timelines of thermal recovery dynamics in patients with SSc. It indicates that even in those with established SSc it is reasonable to repeat cold challenge with a one-hour interval, opening up the possibility of multiple cold challenges on the same day. It also highlights the requirement to enable patients’ gentle movement between imaging sessions to stop decreased perfusion due to sedentary behaviour. It is another step towards designing standard protocols for Clinical Trials of RP treatment.

## Supplementary Information

Below is the link to the electronic supplementary material.

Supplementary Material 1

Supplementary Material 2

## Data Availability

The data underlying this article will be shared on reasonable request to the corresponding author.

## References

[1] Meier, F. M. et al. Update on the profile of the EUSTAR cohort: An analysis of the EULAR scleroderma trials and research group database. *Ann. Rheum. Dis.* **71**, 1355–1360 (2012). 22615460 10.1136/annrheumdis-2011-200742

[2] Kumar, K., Maundrell, A., Proudman, S. Epidemiology of Raynaud’s phenomenon. Raynaud’s phenomenon from pathogenesis to management. In: Wigley FM, Herrick AL, Flavahan NA (eds.). Springer, 2024: 25–40.

[3] Pauling, J. D., Shipley, J. A., Harris, N. D. & McHugh, N. J. Use of infrared thermography as an endpoint in therapeutic trials of Raynaud’s phenomenon and systemic sclerosis. *Clin. Exp. Rheumatol.* **30**, S103–S115 (2012). 22691218

[4] Herrick, A. L. et al. A phase 2 trial investigating the effects of the angiotensin II type 2 receptor agonist C21 in systemic sclerosis-related Raynaud’s. *Rheumatology* **62**, 824–828 (2023). 35894657 10.1093/rheumatology/keac426 PMC9891408

[5] Dziadzio, M. et al. Losartan therapy for Raynaud’s phenomenon and scleroderma: Clinical and biochemical findings in a fifteen-week, randomized, parallel-group, controlled trial. *Arthritis Rheum.* **42**, 2646–2655 (1999). 10616013 10.1002/1529-0131(199912)42:12<2646::AID-ANR21>3.0.CO;2-T

[6] Herrick, A. L. et al. A double-blind, randomized, placebo-controlled crossover trial of the α2C-adrenoceptor antagonist ORM-12741 for prevention of cold-induced vasospasm in patients with systemic sclerosis. *Rheumatology* **53**, 948–952 (2014). 24489014 10.1093/rheumatology/ket421

[7] Selenko-Gebauer, N., Duschek, N., Minimair, G., Stingl, G. & Karlhofer, F. Successful treatment of patients with severe secondary Raynaud’s phenomenon with the endothelin receptor antagonist bosentan. *Rheumatology***suppl_3**, iii45–iii48 (2006). 10.1093/rheumatology/kel290 16987835

[8] Tornling, G. et al. A phase 2 trial investigating the efficacy and safety of the mPGES-1 inhibitor vipoglanstat in systemic sclerosis-related Raynaud’s. *Rheumatology* **30**, keae049 (2024). 10.1093/rheumatology/keae049 PMC11781579 38291895

[9] Coleiro, B. et al. Treatment of Raynaud’s phenomenon with the selective serotonin reuptake inhibitor fluoxetine. *Rheumatology* **40**, 1038–1043 (2001). 11561116 10.1093/rheumatology/40.9.1038

[10] Wilkinson, J., Seibold, J.R., Statistical design and reporting of randomised controlled trials for Raynaud’s phenomenon. Raynaud’s phenomenon from pathogenesis to management. In: Wigley FM, Herrick AL, Flavahan NA (eds.). Springer 2024: 297–307.

[11] Wilkinson, J. D. et al. A multicenter study of the validity and reliability of responses to hand cold challenge as measured by laser speckle contrast imaging and thermography: Outcome measures for systemic sclerosis-related Raynaud’s phenomenon. *Arthritis Rheumatol.* **70**(6), 903–911 (2018). 29457381 10.1002/art.40457 PMC6001804

[12] van den Hoogen, F. et al. 2013 classification criteria for systemic sclerosis: An American college of rheumatology/European league against rheumatism collaborative initiative. *Arthritis Rheum.* **65**, 2737–2747. 10.1002/art.38098 (2013). 24122180 10.1002/art.38098 PMC3930146

[13] Bellando-Randone, S. et al. Progression of patients with Raynaud’s phenomenon to systemic sclerosis: A five-year analysis of the European scleroderma trial and research group multicentre, longitudinal registry study for very early diagnosis of systemic sclerosis (VEDOSS). *Lancet Rheumatol* **3**, e834–e843 (2021). 38287630 10.1016/S2665-9913(21)00244-7

[14] Merkel, P. A. et al. Current status of outcome measure development for clinical trials in systemic sclerosis. Report from OMERACT 6. *J. Rheumatol.* **30**, 1630–1647 (2003). 12858472

[15] Maltez, N. et al. Developing a core set of outcome measure domains to study Raynaud’s phenomenon and digital ulcers in systemic sclerosis: Report from OMERACT 2020. *Semin. Arthritis Rheum.* **51**, 640–643 (2021). 33947582 10.1016/j.semarthrit.2021.04.005

[16] Maltez, N. et al. Domain reporting in systemic sclerosis-related Raynaud’s phenomenon: An OMERACT scoping review. *Semin. Arthritis Rheum.* **61**, 152208 (2023). 37202251 10.1016/j.semarthrit.2023.152208

[17] Pauling, J. D., Reilly, E., Smith, T. & Frech, T. M. Factors influencing Raynaud condition score diary outcomes in systemic sclerosis. *J. Rheumatol.* **46**, 1326–1334 (2019). 30824643 10.3899/jrheum.180818

[18] Merkel, P.A., Herlyn, K., Martin, R.W., Anderson, J.J., Mayes, M.D., Bell, P. et al, Scleroderma Clinical Trials Consortium. Measuring disease activity and functional status in patients with scleroderma and Raynaud’s phenomenon. *Arthritis Rheum*. **46**, 2410–20 (2002) 10.1002/art.10486 12355489

[19] Taylor, L. et al. Impact of season, environmental temperature, and humidity on Raynaud phenomenon in an Australian systemic sclerosis cohort. *Arthritis Care Res* **77**(1), 61–68 (2025). 10.1002/acr.25452 39420564

[20] Virgili-Gervais, G. et al. The association of outdoor temperature and self-reported Raynaud’s phenomenon severity among people with systemic sclerosis: A scleroderma patient-centered intervention network cohort study. *Lancet Rheumatol.* **6**(10), e684–e692 (2024). 39216493 10.1016/S2665-9913(24)00189-9

[21] Dinsdale, G. et al. No seasonal trends in referrals for vascular investigations: Insight into the diagnosis of Raynaud’s phenomenon and systemic sclerosis. *Clin. Exp. Rheumatol.* **42**(8), 1699–1700 (2024). 39152746 10.55563/clinexprheumatol/1eqvih

