# Supplementary material for: Assessing post-cold challenge recovery of thermography as a potential outcome measure in trials of SSc-related Raynaud’s phenomenon
Source: Sci Rep. 2026 May 14;16:22122. doi: 10.1038/s41598-026-50510-5 (PMC13369364; doi:10.1038/s41598-026-50510-5)
Supplement: Supplementary file 1 — Supplementary Material 1 [file 41598_2026_50510_MOESM1_ESM.docx]

**Supplementary Table 1: Demographics of participants, presented as whole cohort, those for whom all fingers recovered, those for whom more than one finger recovered and those for whom no fingers recovered (median [interquartile range, IQR] or number, N (percentage, %)).**

|  |  | All participants  (N=20) | All 8 fingers recovered (N=8) | At least 1 finger recovered  (N=13) | No fingers recovered (N=7) |
| --- | --- | --- | --- | --- | --- |
| **Age (median [IQR]) years** |  | 62 [52-70] | 56 [53-63] | 57 [51-63] | 69 [62-72] |
| **Subtype N (%)** | LcSSc  DcSSc  VEDOSS | 16 (80)  2 (10)  2 (10) | 6 (75)  1 (13)  1 (13) | 10 (77)  2 (15)  1 (8) | 6 (86)  0 (0)  1 (14) |
| **Years since onset of RP (median [IQR]) years** |  | 16 [7-29] | 18 [7-31] | 10 [7-21] | 24 [20-30] |
| **Years since first non-RP clinical manifestation (median [IQR]) years** |  | 10 [4-23] | 7 [3-10] | 10 [4-10] | 24 [14-26] |
| **Colour change N (%)** | W  B  WB  WR  WBR | 2 (10)  1 (5)  5 (25)  1 (5)  11 (55) | 1 (13)  0 (0)  3 (38)  0 (0)  4 (50) | 1 (8)  0 (0)  5 (38)  0 (0)  7 (54) | 1 (14)  1 (14)  0 (0)  1 (14)  4 (57) |
| **Smoker N (%)**  **Previous smoker N (%)**  **Non-smoker N (%)** | | 3 (15)  3 (15)  14 (60) | 2 (25) 2 (25) 4 (50) | 3 (23) 3 (23)  7 (54) | 0 (0) 0 (0) 7 (100) |
| **Immunosuppressants N (%)** | Yes | 5 (25) | 2 (25) | 4 (31) | 1 (14) |
| **Vasodilators N (%)** | Yes | 12 (60) | 4 (50) | 8 (62) | 4 (57) |
| **Previous IV vasodilators N (%)** | Yes | 3 (15) | 2 (25) | 3 (23) | 0 (0) |
| **Debridement N (%)** |  | 0 (0) | 0 (0) | 0 (0) | 0 (0) |
| **Amputation N (%)** |  | 0 (0) | 0 (0) | 0 (0) | 0 (0) |
| **DU last year N (%)** | Yes | 3 (15) | 1 (13) | 1 (8) | 1 (14) |
| **RCS (median [IQR])** |  | 2 [1-4] | 2 [1-4] | 3 [1-5] | 1 [0-2] |

**LcSSc, limited systemic sclerosis; dcSSc, diffuse systemic sclerosis, VEDOSS, very early diagnosis of SSc, RP, Raynaud’s phenomenon; W=white colours in fingers, B= blue colour change in fingers, R=red colour change in fingers; IV, intravenous; DU, digital ulcers; VAS, visual analogue scale; RCS, Raynaud’s condition score [18].**

**Supplementary Table 2: Temperature and timepoint outcome measures pre and post cold challenge (median [interquartile range, IQR]).**

|  | All participants (N=20) | Participants for whom all 8 fingers recovered within 2 hours (N=8) | Participants for whom least 1 finger recovered within 2 hours (N=13) | Participants for whom no fingers recovered within 2 hours (N=7) |
| --- | --- | --- | --- | --- |
| **Temp_base_ ^o^C** | 29.4 [24.8 - 30.6] | 24.7 (23.7-30.5) | 26.8 (24.6-30.5) | 29.6 (28.0-30.3) |
| **DDD ^o^C** | -1.9 (-3.4 - -0.9) | -3.5 (-3.8 - -1.0) | -2.3 (-3.7 - -1.2) | -1.7 (-2.7 - -0.8) |
| **Temp_0_** | 20.9 (20.0-22.8) | 20.2 (20.0-22.2) | 20.4 (19.9-22.4) | 22.6 (20.9-23.1) |
| **Temp_diff_** | 6.4 (4.8 - 8.5) | 4.7 (4.4 - 8.2) | 6.4 (4.7 - 8.8) | 6.4 (6.1 - 8.2) |
| **Temp_max_^o^C** | 29. 5 (25.5 - 32.0) | 31.2 (28.4 – 33.2) | 31.0 (26.7 – 33.1) | 25.5 (25.0 - 29.5) |
| **Temp_f_ ^o^C** | 25.3 (24.1 - 27.8) | 25.4 (25.2 - 28.4) | 25.4 (24.8 – 30.0) | 24.1 (23.0-25.3) |
| **AUC ^o^C*secs** | 1740.7  (1575.5 -  1866.7) | 1734.4  (1574.3 -  1905.9) | 1754.0  (1578.6 –  2006.0) | 1727.4  (1594.1 -  1825.6) |
| **time_25%_ (hh:mm:ss)** | 00:03:58  (00:02:08-  00:08:25) | 00:02:36  (00:01:25-  00:04:22) | 00:02:43  (00:01:32-  00:03:58) | 00:09:06  (00:06:20-  00:27:36) |
| **time_50%_**  **(hh:mm:ss)** | 00:11:41  (00:06:37-  00:22:50) | 00:07:48  (00:05:20-  00:11:19) | 00:08:48  (00:05:42-  00:12:02) | 00:35:43  (00:15:07-  01:59:04) |
| **time_100%_**  **(hh:mm:ss)** | 00:36:46  (00:20:51-  02:00:00) | 00:13:12  (00:08:07-  00:24:39) | 00:31:11  (00:09:45-  00:36:15) | >02:00:00  (02:00:00-  02:00:00) |
| **time_max_**  **(hh:mm:ss)** | 00:48:26  (00:33:35-  01:09:23) | 00:44:20  (00:29:48-  00:56:15) | 00:45:32  (00:33:04-  00:52:24) | 00:52:30  (00:41:52-  01:15:00) |

**Temp_base_, baseline temperature; DDD, distal dorsal difference; Temp_0_, Initial post-cooling temperature; Temp_diff_, difference between baseline and post cooling temperature; Temp_max_, maximum temperature; Temp_f_, final temperature; AUC, area under the recovery curve; time_25%_, time to 25% recovery; time_50%_, time to 50% recovery; time_100%_, time to 100% recovery; time_max_, time to maximum temperature**
