# Supplementary material for: Assessing post-cold challenge recovery of thermography as a potential outcome measure in trials of SSc-related Raynaud’s phenomenon
Source: Sci Rep. 2026 May 14;16:22122. doi: 10.1038/s41598-026-50510-5 (PMC13369364; doi:10.1038/s41598-026-50510-5)
Supplement: Supplementary file 2 — Supplementary Material 2 [file 41598_2026_50510_MOESM2_ESM.pdf]

Supplementary Table 1: Demographics of participants, presented as whole cohort, those for whom all fingers recovered, those for whom more than one finger recovered and those for whom no fingers recovered (median [interquartile range, IQR] or number, N (percentage, %)).

		All participants (N=20)	All 8 fingers recovered (N=8)	At least 1 finger recovered (N=13)	No fingers recovered (N=7)
**Age (median [IQR]) years**		62 [52-70]	56 [53-63]	57 [51-63]	69 [62-72]
**Subtype N (%)**	LcSSc<br>DcSSc<br>VEDOSS	16 (80)<br>2 (10)<br>2 (10)	6 (75)<br>1 (13)<br>1 (13)	10 (77)<br>2 (15)<br>1 (8)	6 (86)<br>0 (0)<br>1 (14)
**Years since onset of RP (median [IQR]) years**		16 [7-29]	18 [7-31]	10 [7-21]	24 [20-30]
**Years since first non-RP clinical manifestation (median [IQR]) years**		10 [4-23]	7 [3-10]	10 [4-10]	24 [14-26]
**Colour change N (%)**	W<br>B<br>WB<br>WR<br>WBR	2 (10)<br>1 (5)<br>5 (25)<br>1 (5)<br>11 (55)	1 (13)<br>0 (0)<br>3 (38)<br>0 (0)<br>4 (50)	1 (8)<br>0 (0)<br>5 (38)<br>0 (0)<br>7 (54)	1 (14)<br>1 (14)<br>0 (0)<br>1 (14)<br>4 (57)
**Smoker N (%)**<br>**Previous smoker N (%)**<br>**Non-smoker N (%)**		3 (15)<br>3 (15)<br>14 (60)	2 (25) 2 (25) 4 (50)	3 (23) 3 (23) 7 (54)	0 (0) 0 (0) 7 (100)
**Immunosuppressants N (%)**	Yes	5 (25)	2 (25)	4 (31)	1 (14)
**Vasodilators N (%)**	Yes	12 (60)	4 (50)	8 (62)	4 (57)
**Previous IV vasodilators N (%)**	Yes	3 (15)	2 (25)	3 (23)	0 (0)
**Debridement N (%)**		0 (0)	0 (0)	0 (0)	0 (0)
**Amputation N (%)**		0 (0)	0 (0)	0 (0)	0 (0)
**DU last year N (%)**	Yes	3 (15)	1 (13)	1 (8)	1 (14)
**RCS (median [IQR])**		2 [1-4]	2 [1-4]	3 [1-5]	1 [0-2]

LcSSc, limited systemic sclerosis; dcSSc, diffuse systemic sclerosis, VEDOSS, very early diagnosis of SSc, RP, Raynaud's phenomenon; W=white colours in fingers, B= blue colour change in fingers, R=red colour change in fingers; IV, intravenous; DU, digital ulcers; VAS, visual analogue scale; RCS, Raynaud's condition score [18].

Supplementary Table 2: Temperature and timepoint outcome measures pre and post cold challenge (median [interquartile range, IQR]).

	All participants (N=20)	Participants for whom all 8 fingers recovered within 2 hours (N=8)	Participants for whom least 1 finger recovered within 2 hours (N=13)	Participants for whom no fingers recovered within 2 hours (N=7)
**$Temp_{base}$ °C**	29.4 [24.8 - 30.6]	24.7 (23.7-30.5)	26.8 (24.6-30.5)	29.6 (28.0-30.3)
**DDD °C**	-1.9 (-3.4 - -0.9)	-3.5 (-3.8 - -1.0)	-2.3 (-3.7 - -1.2)	-1.7 (-2.7 - -0.8)
**$Temp_0$**	20.9 (20.0-22.8)	20.2 (20.0-22.2)	20.4 (19.9-22.4)	22.6 (20.9-23.1)
**$Temp_{diff}$**	6.4 (4.8 - 8.5)	4.7 (4.4 - 8.2)	6.4 (4.7 - 8.8)	6.4 (6.1 - 8.2)
**$Temp_{max}$ °C**	29. 5 (25.5 - 32.0)	31.2 (28.4 – 33.2)	31.0 (26.7 – 33.1)	25.5 (25.0 - 29.5)
**$Temp_f$ °C**	25.3 (24.1 - 27.8)	25.4 (25.2 - 28.4)	25.4 (24.8 – 30.0)	24.1 (23.0-25.3)
**AUC °C*secs**	1740.7 (1575.5 - 1866.7)	1734.4 (1574.3 - 1905.9)	1754.0 (1578.6 – 2006.0)	1727.4 (1594.1 - 1825.6)
**$time_{25\%}$ (hh:mm:ss)**	00:03:58 (00:02:08-00:08:25)	00:02:36 (00:01:25-00:04:22)	00:02:43 (00:01:32-00:03:58)	00:09:06 (00:06:20-00:27:36)
**$time_{50\%}$ (hh:mm:ss)**	00:11:41 (00:06:37-00:22:50)	00:07:48 (00:05:20-00:11:19)	00:08:48 (00:05:42-00:12:02)	00:35:43 (00:15:07-01:59:04)
**$time_{100\%}$ (hh:mm:ss)**	00:36:46 (00:20:51-02:00:00)	00:13:12 (00:08:07-00:24:39)	00:31:11 (00:09:45-00:36:15)	>02:00:00 (02:00:00-02:00:00)
**$time_{max}$ (hh:mm:ss)**	00:48:26 (00:33:35-01:09:23)	00:44:20 (00:29:48-00:56:15)	00:45:32 (00:33:04-00:52:24)	00:52:30 (00:41:52-01:15:00)

$Temp_{base}$, baseline temperature; DDD, distal dorsal difference; $Temp_0$, Initial post-cooling temperature; $Temp_{diff}$, difference between baseline and post cooling temperature; $Temp_{max}$, maximum temperature; $Temp_f$, final temperature; AUC, area under the recovery curve; $time_{25\%}$, time to 25% recovery; $time_{50\%}$, time to 50% recovery; $time_{100\%}$, time to 100% recovery; $time_{max}$, time to maximum temperature
